# Performance and Analysis of Feature Tracking Approaches in Laser Speckle Instrumentation

**DOI:** 10.3390/s19102389

**Published:** 2019-05-24

**Authors:** Thomas Charrett, Ralph Tatam

**Affiliations:** Engineering Photonics, Cranfield University, Cranfield MK43 0AL, UK; r.p.tatam@cranfield.ac.uk

**Keywords:** laser speckle, feature tracking, feature matching, translation measurement, rotation measurement, laser speckle velocimetry

## Abstract

This paper investigates the application of feature tracking algorithms as an alternative data processing method for laser speckle instrumentation. The approach is capable of determining both the speckle pattern translation and rotation and can therefore be used to detect the in-plane rotation and translation of an object simultaneously. A performance assessment of widely used feature detection and matching algorithms from the computer vision field, for both translation and rotation measurements from laser speckle patterns, is presented. The accuracy of translation measurements using the feature tracking approach was found to be similar to that of correlation-based processing with accuracies of 0.025–0.04 pixels and a typical precision of 0.02–0.09 pixels depending upon the method and image size used. The performance for in-plane rotation measurements are also presented with rotation measurement accuracies of <0.01° found to be achievable over an angle range of ±10° and of <0.1° over a range of ±25°, with a typical precision between 0.02 and 0.08° depending upon method and image size. The measurement range is found to be limited by the failure to match sufficient speckles at larger rotation angles. An analysis of each stage of the process was conducted to identify the most suitable approaches for use with laser speckle images and areas requiring further improvement. A quantitative approach to assessing different feature tracking methods is described, and reference data sets of experimentally translated and rotated speckle patterns from a range of surface finishes and surface roughness are presented. As a result, three areas that lead to the failure of the matching process are identified as areas for future investigation: the inability to detect the same features in partially decorrelated images leading to unmatchable features, the variance of computed feature orientation between frames leading to different descriptors being calculated for the same feature, and the failure of the matching processes due to the inability to discriminate between different features in speckle images.

## 1. Introduction

Laser speckle pattern correlation is a measurement technique using the translation and decorrelation of recorded laser speckle patterns to infer information about the motion of the illuminated object. This approach can be used to measure object translation, rotation, and strain [1,2], and recently there has been increased interest in the technique for practical applications in industry and robotics [3,4,5,6,7,8]. Laser speckle has been applied to robotic tool speed sensing [6] and positioning [8], offering potentially higher accuracies (<0.1 mm) than using the robot joint encoders alone (typically 0.5–3 mm). Here, it offers a potentially lower cost and more widely applicable solution than the use of expensive external laser trackers/interferometers, which can offer similar levels of positioning performance ( 0.1 mm) but require a continuous line-of-sight [9]. In both industrial robotics and robotic navigation using speckle odometry [4,7], the ability to simultaneously measure the in-plane rotation, something that is not currently possible using conventional correlation processing, would allow greater positioning accuracy via reduction of Abbe errors, as well as additional capability. Hence, there is interest in new approaches offering the potential for simultaneous measurement of both the translation and rotation of the speckle pattern, and in the measurement performance achievable.

Practical implementations of in-plane rotation measurement utilising the rotation of laser speckle patterns for the detection of in-plane rotation are few. Saleh [10] used the value of the correlation coefficient to determine rotation; however, this is not practical in situations where decorrelation can occur due to other factors such as translations. While Wang et al. [11] proposed an interferometric speckle approach to in-plane rotation measurement, this is too complex and bulky for many applications. The Fourier–Mellin transform image processing technique [12] can been used to detect in-plane image translation and rotation simultaneously, and has been reported for use in robotic vehicle odometry [13]. However, this approaches uses imaged surface features rather than laser speckle patterns and hence requires features to be present, unlike laser speckle, which can be formed from most surfaces. The Fourier–Mellin transform is also relatively slow, requiring a 2D FFT to ensure translation invariance followed by image re-sampling to log-polar coordinates to find rotation angles and a further transformation to remove the rotation before finding translation by correlation.

This paper investigates the application of an alternative data processing method for laser speckle instrumentation that mirrors the human approach used in viewing speckle patterns, i.e., identifying and tracking characteristic speckles present in both images to determine the speckle shift and rotation. Although such feature detection and tracking is a well-developed concept in computer vision, their application in laser speckle sensing is limited. To our knowledge, the only prior application of feature matching with laser speckle patterns is the use of the scale invariant feature transform (SIFT) algorithm [14] for personal identification card recognition [15,16] and a preliminary study of the techniques presented here [17]. For application to identification card recognition [15,16], a feature matching approach was used to ensure that speckle patterns can be matched to a database of speckle patterns even in the presence of translations and rotations; however, no quantitative measurements of these was necessary. While in [17] we presented preliminary quantitative measurements, we did not explore fully the performance limitations in depth. Such feature tracking approaches are also potentially well suited to speckle positioning sensing for industrial and robotics applications, allowing not only the speckle translation to be determined but also the object rotation to be computed. A further benefit is the potential for allowing data reduction, in approaches utilising a database of laser speckle patterns, with the storage of these reference speckle patterns as a sequence of speckle descriptors rather than full image data.

The remainder of this paper is structured as follows: Section 2 gives an overview of the feature matching process. Section 3 details the methods used in this study, with a description of the some common feature detection and description methods and the implementations that are used in this study in Section 3.1 and Section 3.2. In Section 3.3, the acquisition of the experimental laser speckle patterns used to assess the methods is described, including a set of reference speckle patterns from different surface finishes with applied translations and rotations. In Section 4, the applicability of the feature tracking approaches to the processing of laser speckle images is investigated. The translation performance is compared with conventional correlation-based speckle processing and rotation performance assessed. Finally, in Section 5, an in-depth investigation is presented in which each stage of the feature tracking process is examined individually with the aim of identifying the approaches most suited to laser speckle pattern processing and areas requiring improvement and further development.

## 2. Overview of Feature Matching Process

The feature tracking process can be divided into three stages: feature detection, feature description and matching, and calculation of the transformation describing the translation and rotation of the speckle pattern between the two images. In the first stage, feature detection, points of interest are automatically determined within the images to be matched. This is then followed by a feature description stage where some form of descriptor/identifier for each feature is calculated using the surrounding pixels. As a preliminary step, in many methods it is often necessary to define a feature orientation that is used to guide this descriptor calculation. These descriptors are then used to match features between the images via the calculation of a distance measure. The final stage in the processing is to use the features/speckles that have been matched between two images to determine the translation and rotation of the speckle pattern, i.e., to find the transform, *T*, that relates a set of points *P* detected in the first image to a set $P^{\prime }$ in the second, given by [18]:

(1) $$\begin{array}{c} TP=P^{\prime } \end{array}$$

(2) $$\begin{array}{c} \left[\begin{array}{ccc} \cos\theta , & -\sin\theta , & A_{x}\\ \sin\theta , & \cos\theta , & A_{y} \end{array}\right]\left[\begin{array}{c} x\\ y\\ 1 \end{array}\right]=\left[\begin{array}{c} x^{\prime }\\ y^{\prime } \end{array}\right] \end{array}$$

where $A_{x},A_{y}$ is the speckle translation, and θ is the rotation. This can be solved provided there are sufficient matched points using the least squares approximation of the system to find the rotation angle and translation at the (0,0) image position, i.e., top-left corner:

(3) $$\begin{array}{c} \left[\begin{array}{cccc} x_{1}, & -y_{1}, & 1, & 0\\ y_{1}, & x_{1}, & 0, & 1\\ x_{2}, & -y_{2}, & 1, & 0\\ y_{2}, & x_{2}, & 0, & 1\\ \vdots & \vdots & \vdots & \vdots \\ x_{n}, & -y_{n}, & 1, & 0\\ y_{n}, & x_{n}, & 0, & 1 \end{array}\right]\left[\begin{array}{c} \cos\theta \\ \sin\theta \\ A_{x}\\ A_{y} \end{array}\right]=\left[\begin{array}{c} x_{1}^{\prime }\\ y_{1}^{\prime }\\ x_{2}^{\prime }\\ y_{2}^{\prime }\\ \vdots \\ x_{n}^{\prime }\\ y_{n}^{\prime } \end{array}\right]. \end{array}$$

## 3. Methods

### 3.1. Feature Detection Methods

The first stage of the processing involves the identification of the speckles or features to be tracked. In this work, some common feature detectors, with implementations available in the OpenCV [19] library, were selected for investigation. These can be grouped into three related families of approaches: intensity gradient approaches such as Harris corners [20], so-called accelerated segment tests (AST) methods [21,22], and blob detection algorithms using difference of Gaussians [23]. A summary of the feature detection methods used is shown in Table 1 along with the parameters used and details of their implementation using the OpenCV python bindings. These values were determined by a trial and error approach using example speckle patterns or by using suggested or typical values found in the OpenCV documentation.

The Harris corner detector [20] and related Shi–Tomasi method [22] (also known by the acronym GFTT—Good Features To Track) are based on a calculation of the local intensity gradients within a neighbourhood of every pixel. Here the term corner is used to represent a feature with large intensity gradients which for this application can be viewed as individual speckles provided the speckle size is small, typically around 2–3 pixels in diameter. The intensity gradients within the neighbourhood of a pixel can be described by the matrix:

(4) $$M=\left[\begin{array}{cc} I_{x}^{2} & I_{x}I_{y}\\ I_{x}I_{y} & I_{y}^{2} \end{array}\right]$$

where $I_{x}$, $I_{y}$ are partial derivatives of the pixel intensity in the *x* and *y* directions, and the summation is performed over a circular window in the pixel neighbourhood that has been smoothed with a Gaussian kernel. If both eigenvalues $\lambda_{1}$ and $\lambda_{2}$ of the matrix *M* are high, then the point is classified as a corner. However, calculation of the eigenvalues is computationally expensive, so the following is used as a measure of the corner response [20]:

(5) $$\begin{array}{cc} R & =det(M)-\kappa trace(M)\\ R & =\lambda_{1}\lambda_{2}-\kappa \left(\lambda_{1}+\lambda_{2}\right)\\ R & =\left(I_{x}^{2}I_{y}^{2}-I_{x}^{2}I_{y}^{2}\right)-\kappa \left(I_{x}^{2}+I_{y}^{2}\right) \end{array}$$

κ is the Harris free parameter, the value of which has to be determined empirically and is typically 0.04–0.15. *R* is positive in the corner region, negative in the edge regions, and small in flat regions [20]. Alternatively, Shi and Tomasi [22] proposed the criteria:

(6) $$R=min\left(\lambda_{1},\lambda_{2}\right)>\lambda_{min}$$

where $\lambda_{min}$ is a threshold determining whether a pixel is a flat region, an edge, or a corner.

The second class of detectors are the accelerated segment tests (AST) methods include the FAST (Features from Accelerated Segment Test) detector [21] and the AGAST (Adaptive and Generic Accelerated Segment Test) corner detector [24]. These operate using a simple concept based upon the examination of pixels on a ring around the point of interest with a corner detected when more than *n* contiguous pixels on the ring are all either significantly darker or brighter than the central point. The ring can be a circle of 8, 12, or 16 pixels, and typically n=9 for a 16 pixel ring [21]. Here both methods were used with a 16 pixel ring. This process is then optimised using a machine learning approach to generate an efficient decision tree [21]. The AGAST (Adaptive and Generic Accelerated Segment Test) corner detector [24] modifies the decision trees to provide high performance for arbitrary environments without training. For both methods, the optional non-maximal suppression [21] was applied to remove points describing the same corner, as this was found to have minimal impact on the processing time of the feature detection stage whilst limiting the detected corners to those with the best corner response. Many widely used combined feature detection and description algorithms, such as ORB [25] and BRISK [26], use FAST-like detectors. The ORB algorithm uses an orientated version of the FAST (oFAST) in the detection stage using the vector between the feature position and the intensity centroid of the surrounding image patch to define the orientation. In BRISK, comparisons between the gradients between long range pairs of points surrounding the feature are used.

An alternative to seeking corner features is to look for so-called blobs where the image is segmented based upon intensity, colour, or texture. The commonly used combined feature detection and matching algorithms Scale-Invariant Feature Transform (SIFT) [14] and Speeded-Up Robust Features (SURF) [27] both use a Difference of Gaussians (DoG) approximation to detect blobs. However, SURF uses a more efficient algorithm where the DoG can be approximated directly using a set of box filters instead of repeatedly filtering the image with Gaussian filters at different scales. This together with the use of integral images in the application of the box filters yields significantly lower processing times. The SURF method also computes the orientation of feature using a pair of Haar wavelets [27]. This can be skipped for the so-called Upright SURF (USURF) method and both methods where tested.

### 3.2. Feature Description and Matching Methods

In the feature matching stage, the detected features must be identified and matched between images by some form of feature descriptor. In this work, four combined detection and description methods with available implementations in the OpenCV library were investigated for use with speckle patterns: USURF [27], SURF [27], ORB [28], and BRISK [26]. A summary of the feature matching methods used is shown in Table 2 along with the parameters used. Methods including options for detecting features of different scales were disabled to speed computation, as no speckle scale change detection is anticipated or required.

The SURF and USURF methods [27] use a descriptor calculated using an image patch (20 × 20 pixels), rotated accordingly to the computed orientation for the SURF method. This square is divided into a 4 × 4 grid of sub-regions, and in every sub-region the horizontal ($d_{x}$) and vertical ($d_{y}$) Haar wavelet responses at 5 × 5 regularly spaced sample points are calculated. For each sub-region a 4D descriptor vector $v=(\sum d_{x},\sum d_{y},\sum |d_{x}|,\sum |d_{y}|)$ is constructed based upon the underlying region, giving a total feature descriptor length of 64 values. The USURF method omits the orientation step and can maintain a robustness to rotation of about ±15° [27]. An extended form computes additional sums by dividing the above sums into cases when $d_{x,y}<0$ and $d_{x,y}\geq 0$, giving an 8D vector for each sub-region and a total feature descriptor length of 128 values.

The ORB (Orientated FAST and Rotated BRIEF) algorithm uses a BRIEF [28] (Binary Robust Independent Elementary Features) like descriptor that constructs a bit string description of an image patch using a series of binary intensity tests between pixels pairs in the neighbourhood of the feature. The Rotated BRIEF descriptor introduced in ORB uses orientation information from the feature detection stage to “steer” the pairs used and produces a bit string of 256 point comparisons taken from an image patch size around the feature (default patch size of 64 × 64 pixels).

The final feature descriptor method investigated was BRISK (Binary Robust Invariant Scalable Keypoints) this uses a modified BRIEF descriptor where the random pixel-pair selection has been changed to an organised procedure with points positioned on concentric circles around the feature. Long-range pairing are used for defining the orientation of features, and short-range pairings are used for descriptor encoding, producing a bit string of 512 point comparisons from an image patch surrounding the feature.

After the feature descriptors have been computed, a brute force matching algorithm that finds the closest matching key-point descriptor in the second set is employed by trying each one and computing the distance based upon either the L1-norm (sum of the absolute differences $\Sigma |d_{1}-d_{2}|$) or the L2-norm (sum of the squared differences $\Sigma {\left(d_{1}-d_{2}\right)}^{2}$), where $d_{1}$ and $d_{2}$ represent the descriptor vectors to be compared. Methods that use a binary descriptor, such as ORB and BRISK, use the Hamming distance (the number of ones in the bitwise XOR comparison $d_{1}⊕d_{2}$) for fast comparison between the descriptors. It should be noted that the ORB and BRISK implementations in OpenCV use different bit length descriptors as specified by the authors of these algorithms; the ORB algorithm uses a 256 bit descriptor [25], while BRISK uses a 512 bit descriptor [26]. An additional filtering step can then be used in an attempt to remove incorrect matches before the calculation of the transform by application of a distance threshold above which matches are excluded. The level of this threshold was determined by trial and error; however, appropriate thresholds can be chosen using the approach proposed later in Section 5.5.3.

### 3.3. Experimental Methods

To investigate the performance of the feature tracking approaches, experimental speckle patterns were acquired using a speckle velocimetry sensor [6], consisting of a 658 nm fibre coupled diode laser (FibreTec II FTEC2658) together with a camera (Ximea MQ013CG-ON, 1280 × 1024 pixels, 4.8 µm pixel size) positioned at a distance of 150 mm from the illuminated surface. The laser output was expanded to a spot of ∼8 mm diameter, and as no imaging lens was used the resulting objective speckle patterns had a speckle size diameter of ∼4 pixels. The surface was mounted on a six degree-of-freedom translation stage (ALIO Hybrid-Hexapod AI-HYBRID-HEX-60XY-15Z-56R) that could be used to apply controlled translations and rotations.

**Figure 1:**
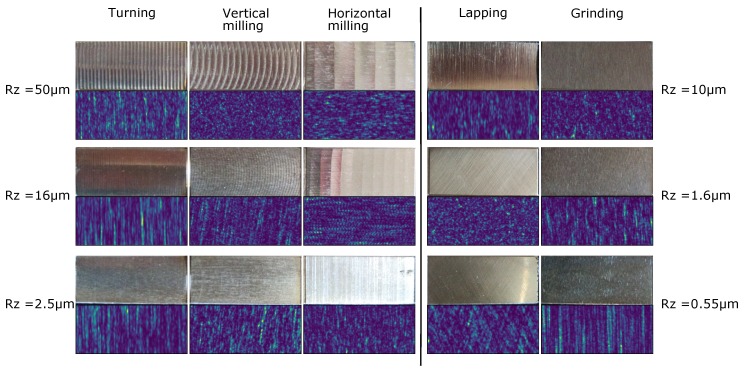
Surface finish samples with various surface roughnesses $R_{z}$ used together with a 250 × 100 pixel sub region of the resulting speckle pattern for each sample.

As different materials and surface finishes can result in speckle patterns with significantly different appearances, the surface material used was varied. A cast aluminium plate (approximately 220 × 220 mm in dimension) was used to test continuous translations requiring longer displacements, and additional data sets were acquired from samples prepared with different surface finishing techniques and finished to different surface roughnesses. These samples consisted of a composite set of surface roughness specimens (Robert & Co., Ltd., England). Each sample was 25 × 10 mm in dimension, and the surfaces and resulting speckle patterns can be seen in Figure 1. Data sets consisting of a stepped translation between 0 and 2.8 mm in 50 µm steps and stepped rotations between 0 and 360° in 5° steps were acquired for each sample. A summary of the experimental data sets acquired is shown in Table 3 and are available from the Cranfield University data repository.

## 4. Initial Performance Assessment of Feature Tracking Approaches

An initial assessment of the performance achievable was conducted using the feature detection and matching algorithms USURF, SURF, ORB, and BRISK, described in Section 3.2. These are combined feature detection and description methods that were implemented in Python using the OpenCV library [19], and the details are summarised in Table 2. In this initial assessment, the methods were compared with the normalised cross-correlation approach commonly used for translation measurement. Processing times and image size requirements were investigated, and the achievable rotational accuracy and measurement range were assessed.

### 4.1. Translation Performance

**Figure 2:**
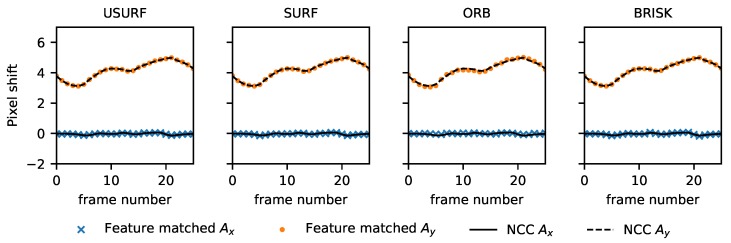
Comparison between experimental speckle velocimetry data processed using the normalised cross-correlation (NCC) and feature tracking methods. The speckle shift found using the NCC method is shown by the solid and dashed lines for the *x* and *y* components, respectively. The feature tracking results are shown as the data points, crosses, and dots for the *x* and *y* components, respectively.

**Figure 3:**
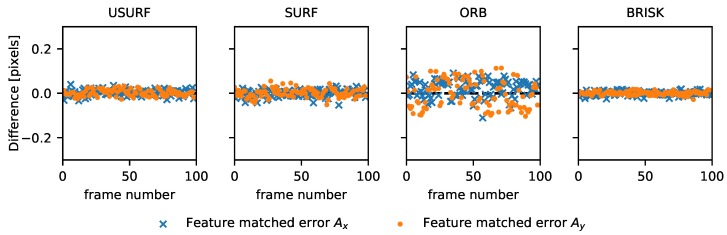
Difference between experimental speckle velocimetry data processed using the normalised cross-correlation (NCC) and each of the feature tracking methods. Here the crosses and dots represent the *x* and *y* components, respectively.

In the first experiment, the cast aluminium plate was used as a surface with a continuous translation in the x-direction at ∼5 mm/s applied. A set of 100 512 × 512 images were acquired at 500 fps (Data Set 1, Table 3), and the resulting images were then processed using the methods described in Table 2 to find the translation and rotation between consecutive frames. This was compared to processing using a normalised cross-correlation to determine the translation between frames, as is currently done in speckle velocimetry [4]. The results are shown in Figure 2, with the *x* and *y* speckle shift for the feature tracking methods shown by the data points, and those using the normalised cross-correlation method are shown by the solid and dashed lines for the *x* and *y* components, respectively. In Figure 3, the difference between these two methods is shown. It can be seen that there is good agreement between the normalised cross-correlation and feature tracking results with the BRISK method performing best with differences of <0.02 pixels between the two. This is likely due to the larger number of features matched using this method (see Table 4 for a comparison of the number of matched features for each method).

**Figure 4:**
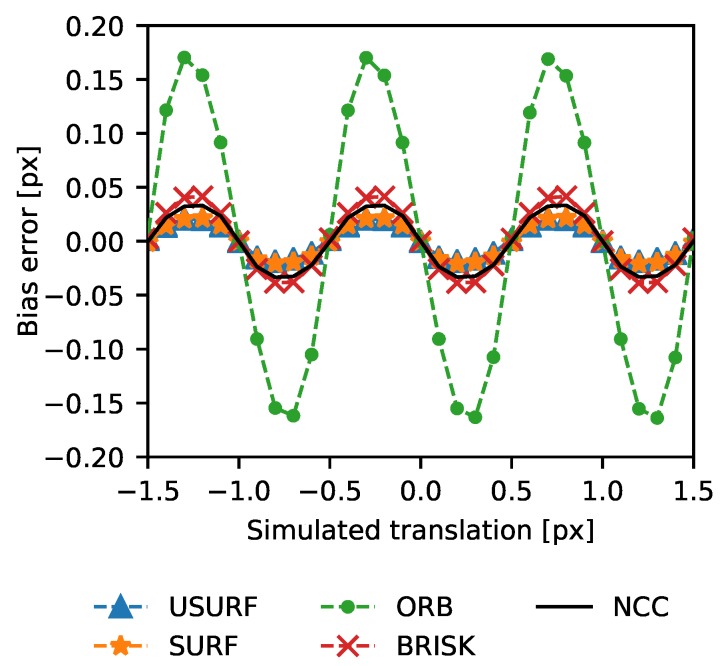
Accuracy of translation measurements made using the ORB, BRISK, USURF, and SURF feature tracking methods, using artificially translated speckle patterns to show the remaining pixel locking effect. The normalised cross-correlation with a 3-point Gaussian peak fitting results are shown for comparison.

To further assess the accuracy of the feature tracking approach, artificially shifted patterns where generated using a set of uncorrelated speckle patterns (512 × 512 pixels) captured from different physical positions on the aluminium plate (Data Set 2, Table 3). A window in each image of this set was artificially translated by sub-pixel shifts, and the translation was calculated using the feature tracking methods as above. Sub-pixel translations in 0.1 pixel increments was achieved by first magnifying the image by a factor of 10, shifting the window and then de-magnifying via binning of the pixel intensities. The results are shown in Figure 4, where the bias error or accuracy (calculated as the mean calculated translation minus the applied translation) is shown for a range of simulated translations for the feature tracking methods, together with those for the normalised cross-correlation (NCC) with 3-point Gaussian peak fitting method for comparison. It can be seen that all methods including the normalised cross-correlation exhibit pixel-locking effects [29]. The BRISK algorithm has pixel-locking effects of ∼1/20 of a pixel similar to those exhibited by the NCC reference method and to those previously reported for correlation-based methods [29]. However, both SURF and USURF perform better than the NCC in this respect with bias errors of <∼0.025 vs. ∼0.033 pixels for the NCC method. Both SURF and USURF methods perform similarly as expected as they are essentially the same when there is zero rotation present. The ORB method performs significantly worse than the other methods with large bias errors of up to 0.2 pixels, suggesting a cause for the larger difference seen when comparing this method with the NCC results in Figure 3. This may be due to the integer pixel location of key points in the method as opposed to sub-pixel locations used in the other methods.

### 4.2. Rotation Performance

**Figure 5:**
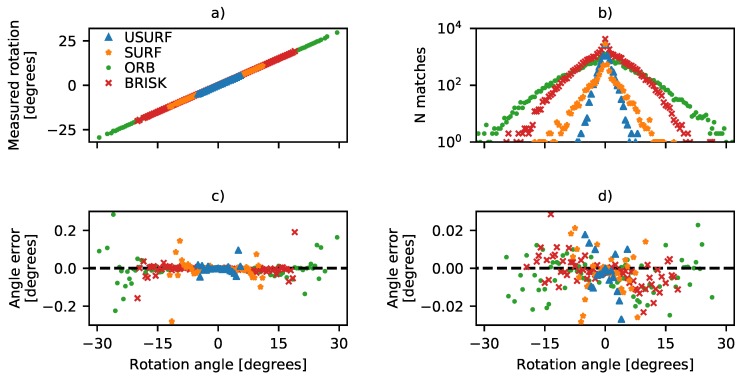
Accuracy of rotation measurements made using the USURF, SURF, ORB, and BRISK feature tracking methods, using experimentally rotated speckle patterns. (**a**) Measured rotation versus applied rotation. (**b**) The number of matched features after application of distance threshold, described in Section 4.2 and Table 2. Missing data points are where too few features were matched to successfully calculate the transform. The remaining plots (**c**,**d**) show the measurement error, with (**d**) having the vertical scale changed to show achievable accuracy.

Next, the accuracy achievable for measurements of speckle pattern rotation was assessed in a similar way using a sequence of speckle patterns recorded from the cast aluminium plate (Data Set 3, Table 3). The surface was rotated at angles between 0 and 360 degrees in 0.5 degree steps using the rotation capabilities of the stage (repeatability ±0.5 arc-second and 0.04 arc-second resolution). The captured images were then processed as before, and the results are shown in Figure 5. Here, (a) shows the measured versus applied rotation for the angles between 0 and 30°, (b) shows the number of features matched versus the rotation angle for each of the methods. The final plots in Figure 5c,d show the error in measured angle (i.e., the difference between the applied and measured angles of rotation) plotted with differing y-axis limits. From this, it can seen that feature tracking methods can be successfully used to measure rotation; however, there are significant differences in the performance of the different matching techniques. The SURF descriptors can be seen to perform the worst, with the non-orientated version (USURF) failing first after ±5°. This could be expected, as this method includes no orientation of the feature descriptor; however, it appears to fail much sooner than the ±15° suggested in [27]. The orientated version (SURF) performs slightly better but again fails after ±10°, while the BRISK method performs slightly better with measurements possible up to ±20°, and the ORB method is the best performing with reliable calculations of rotation over the range ±25°. This can be explained by the number of successfully matched features, where the ORB method has significantly more matches than the other methods at larger angles, as shown in Figure 5b. At larger angles, with fewer matching features, the error can be seen to increase. The error in the rotation angle increases from <0.02° at angles <5° to 0.2° at ∼20° before finally there are insufficient points to solve the transform (Equation (3)) and no measurement is possible. In this measurement, a distance threshold was applied as shown in Table 2 to remove incorrect matches and prioritise the integrity of the results over potentially spurious measurements. At angles >30°, too few matches were made, and no measurement could be made; however, relaxing the distance threshold measure led to incorrect matches and spurious measurements and increased error even at lower rotation angles. This suggests that higher accuracies at larger angles are possible if sufficient high-quality feature matches can be found.

### 4.3. Processing Time, Measurement Precision, and Image Size

In many applications, it is necessary to process the speckle patterns rapidly [4,6]; hence, often only a small area or window of the available speckle pattern is processed. Similarly, to ensure maximum correspondence between the speckle patterns to be matched, a smaller window is often used with an offset between the window position in the two images [6]. The influence of changing the size of this processed window on the processing time is summarised in Table 4, where the results of processing Data Set 1 (continuous translation) and Data Set 3 (stepped rotations) are shown using different size windows in the images. The processing times, especially at small window sizes, are acceptable for many applications; for example, for speckle velocimetry an update rate of 50 Hz or 20 ms per frame pair is acceptable. For comparison an optimised implementation of the normalised cross-correlation has processing times on the same CPU of 0.25 ms for a 128 ×128 pixel window and 6.3 ms for a 512 × 512 pixel window; however, this provides no rotation information. Also shown in Table 4 is the influence on the achievable measurement precision. Here the $\sigma_{A_{x}}$, $\sigma_{A_{y}}$, and $\sigma_{\theta }$ are the standard deviations of the errors calculated via comparison with normalised cross-correlation results and zero rotation for the translation measurements and by comparison with applied stage rotation for the rotation measurements. It can be seen that, even at small window sizes, the methods show good agreement; however, with significantly larger errors, especially for the ORB method. This may be explained by the low number of matched speckles compared to the other methods. However, the SURF method has a similar number of matches but only shows a slight increase in the errors, perhaps suggesting a higher percentage of good or valid matches. For the rotating data set, changing the window size had a significant impact due to fewer matches; with the ORB method, using a 128 ×128 pixel window limits the measurement range to ±15° and increases the error in the calculated rotation angle ∼×10 to ±0.2°, compared to the results shown in Figure 5 for a 512 × 512 image. Again, these results suggest that further investigation into optimising the number of matched speckles and the number of valid matches is required to make performance improvements.

## 5. Analysis of Individual Feature Tracking Stages

The initial investigations above suggest that the feature tracking method could potentially be improved by optimisation of the number of features successfully matched. This would provide a larger angular measurement range and lower errors, and potentially allow for smaller image sizes to be used and reduced CPU processing load. To investigate this, an analysis of each individual stage of the matching process was performed to determine the most appropriate methods for use with laser speckle patterns and identify areas requiring improvements.

### 5.1. Analytical Methods

**Figure 6:**
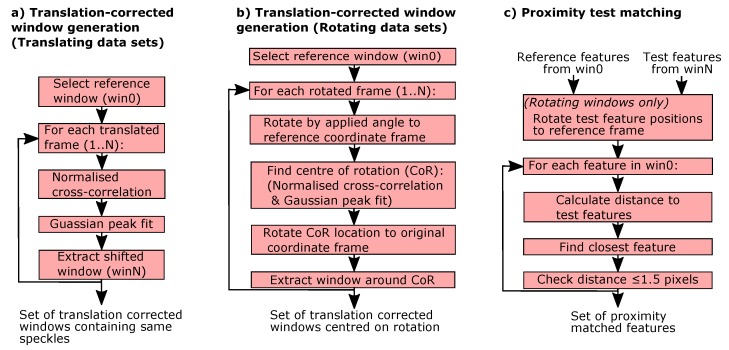
Flow diagrams showing the analytical methods used to investigate the performance of feature tracking algorithms. (**a**) The generation of translation correction windows containing common speckles for translating data sets. (**b**) The generation of translation correction windows from rotating data sets. (**c**) The proximity test method of matching speckles between windows.

To allow a systematic investigation of the performance of the different feature tracking algorithms, several methods are used throughout this analysis. To enable the investigation of the influence of translation on the performance of the different methods, a process was developed to extract a set of translation-corrected windows from a data set. In this way, any translation is removed and the same speckles should be present in all windows, at the same location, with only inter-frame changes such as decorrelation and sampling differences remaining. The procedure, shown in Figure 6a, was to initially extract a reference window from the first image in the set. Normalised cross-correlation [30] together with Gaussian peak fitting [29] is then used to find the position of the same window in each subsequent frame. This translation-corrected window is then extracted from the image to the nearest whole pixel. The pattern translation in *x* and *y* is also recorded together with the correlation co-efficient of the two windows overlaid, providing a measure of the speckle pattern decorrelation or change with translation.

To similarly enable the investigation of the influence of rotation, a process was developed to extract a set of translation-corrected windows from a rotating data set, as shown in Figure 6b. These windows are centred upon the rotation and contain a consistent set of speckles, again with only inter-frame changes such as decorrelation effects and sampling variations present. To achieve this, each frame was first rotated digitally about an approximate centre-of-rotation by its known rotation with respect to a reference frame as determined by the commanded stage rotation. Any remaining translation or error in the centre-of-rotation estimate was then found using normalised cross-correlation and Gaussian peak fitting, and finally a window about this refined centre-of-rotation in the original frame was then extracted. Videos of these translation corrected windows for both translating and rotating speckle patterns are available in the Supplementary Materials.

The third method used throughout is the use of proximity testing to find a set of matching features between the translation-corrected windows generated using the methods described above. In this proximity test, shown in Figure 6c, the positions of features between the reference window and subsequent windows are compared, with a matching feature said to be found if there is a feature within a ±1.5 pixel radius. This ensures that the same features in the two images are being compared and allows features to be matched in a way that is independent of the performance of any of the matching scheme described in Section 3.2. The small radius used allows features to be matched even if the feature detector has marked a slightly different position due to camera pixel sampling variations or image noise and allows for consideration of sub-pixel errors introduced due to the integer pixel limitation in locating the window. A similar approach is used to match features in the rotating windows, although here the features will not have the same positions in the windows due to the rotation. Hence, the feature positions are first rotated by the known angle around the centre of the window to find their position in the reference window frame before the proximity test above is applied. Additionally, a circular mask is implemented to remove features in the corners of the windows, which are not always present due to the rotation. This is implemented by excluding features lying outside of a defined radius from the centre of the window from the analysis.

### 5.2. Feature Detection Speed

Initially the speed of the detectors was investigated, as this is the critical parameter for application in manufacturing and robotics, where high speed processing is essential. Data Set 2 (Table 3) was processed using the different feature detection methods described above, and the results are shown in Table 5. Here it can be seen that the accelerated segment test type detectors, such as the FAST and AGAST, are the fastest. The additional orientation calculations in methods such as the orientated FAST methods used in ORB and BRISK, and the DoG/SURF detectors, can be seen to increase the processing time significantly, with the ORB detector approach performing the best in terms of processing speed.

### 5.3. Feature Detection Robustness

The second measure used to assess the suitability and performance of the different feature detection methods is the robustness—the ability to detect the same feature/speckle in multiple frames despite frame-to-frame variations in the recorded speckle pattern. Such variations may exist due to a number of reasons: changing speckle shapes/intensities due to the decorrelation of the speckle field due to object translations and rotations, differences in the sampling of the speckle pattern at the detector, changing illumination intensity due to laser power fluctuations, and the addition of random camera noise. The robustness $R_{detect}$ can be described by the ratio, $R_{detect}=N_{m}/N_{0}$, i.e., the proportion of features that are detected in both frames, where $N_{m}$ is the number of feature detected/matched in both windows, and $N_{0}$ is the number originally detected in the reference image/window. The robustness of the detector will be important to ensure that the maximum number of features can be matched for a given image size, allowing for increased measurement range and accuracy, and to minimise computation cost in the description and matching stage where excess calculations will be required if features are not detected similarly between frames.

The detector robustness to translation was assessed using Data Set 4 in Table 3—a sequence of 50 µm stepped translations between 0 and 2.8 mm for various surface finishing techniques and surface roughness resulting in speckle translations in the *x* (horizontal) image direction of 0 to ∼1200 pixels. Initially, a set of translation-corrected windows are extracted from each data set using the method described in Section 5.1. A window size of 128 × 960 (width, height) pixels was used to maximise the number of features present in the windows, while also allowing the full range of translations in the horizontal direction to be investigated. Next, features are detected in each window using the methods shown in Table 1, and these are then compared to the features found in the reference window using the proximity matching approach (see Section 5.1) to determine if the same feature is detected in both. The detection ratio for a given pattern translation can then be calculated.

**Figure 7:**
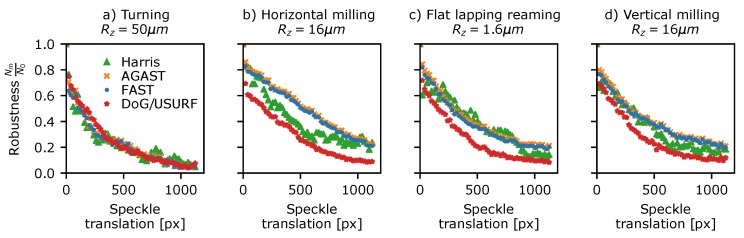
Detector translational robustness plotted against measured speckle translation for a selection of surface treatments shown in Figure 1. (**a**) Turning $R_{z}$ = 50 µm, (**b**) horizontal milling $R_{z}$ = 16 µm, (**c**) flat lapping reaming $R_{z}$ = 1.6 µm, and (**d**) vertical milling $R_{z}$ = 16 µm.

The results are shown in Figure 7 plotted versus speckle translation for a subset of the surfaces shown in Figure 1. Here, the ORB, Brisk, and SURF detectors have been omitted for clarity, as these are essentially orientated versions of the FAST and DoG methods and give similar or identical results. All of the surfaces showed similar results (see Supplementary Materials for plots for all surfaces) with a large drop in the number of features being detected in both windows as the translation and pattern decorrelation increases. The AST-like detector methods (FAST and AGAST) consistently perform the best throughout all samples with the exception of surfaces finished by turning where all methods performed comparably. Typically the detector robustness drops to 25–30% after a ∼500 pixel shift, which represents a pattern correlation co-efficient of C≈0.6 (where C=1 is a perfect correlation and C=0 indicates a completely decorrelated speckle pattern).

**Figure 8:**
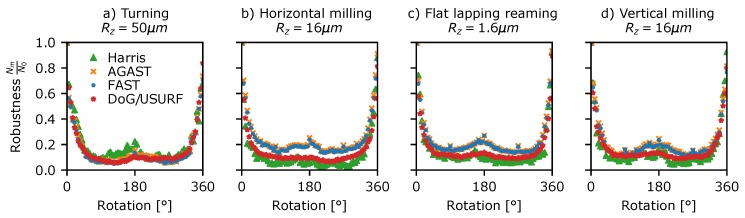
Detector rotational robustness plotted against rotation angle for a selection of surface treatments shown in Figure 1. (**a**) Turning $R_{z}$ = 50 µm, (**b**) horizontal milling $R_{z}$ = 16 µm, (**c**) flat lapping reaming $R_{z}$ = 1.6 µm, and (**d**) vertical milling $R_{z}$ = 16 µm.

A similar approach was used to investigate the robustness of the detector to rotating speckle patterns. This was assessed using Data Set 5 (Table 3) consisting of stepped rotations of 5° from 0 to 360° for each of the surfaces in Figure 1. This data was processed in a similar way to the translating speckle patterns above, using circularly masked windows centred upon the rotation and the modified proximity test, where the image coordinates of each detected feature are first rotated to the reference image’s coordinate-frame before matching. The results, as shown in Figure 8, show that, as with translation, the fraction of features detected in both frames drops rapidly with rotation of the pattern, reaching 50% by ∼12° and 10–20% by 90°. Again, the AST-like detectors appear most consistent in detecting the same features. Interestingly, there is a slight peak in the detection at 180° rotation, suggesting that the sampling grid of the camera pixels may have some influence on which features are identified in the speckle pattern. Similar plots of detector robustness to translations and rotations for the other surface samples shown in Figure 1 can be found in the Supplementary Materials.

**Figure 9:**
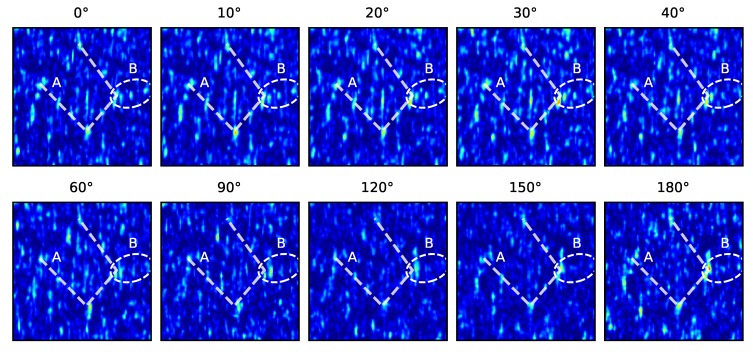
Rotation- and translation-corrected speckle patterns taken from the horizontal milling $R_{z}$ = 2.5 µm sample at various rotation angles showing the change in the pattern with rotation. Label A (shown by connecting lines) shows a fixed pattern of speckles identifiable throughout, and B (ellipse) shows a group of speckles that appear fade/vanish at higher rotations.

Figure 9 shows some examples of a translation- and rotation-corrected speckle pattern taken from the horizontal milling $R_{z}$ = 2.5 µm sample, to illustrate the changes in the pattern with rotation. From this, it can be seen that some features/speckles are present throughout the rotation (indicated by Label A and dashed connecting lines in Figure 9). However, some speckles also appear to change significantly, e.g., the cluster of speckles highlighted by the ellipse/Label B, where some speckles fade/vanish at higher rotations. Some of these differences may be explained by the different sampling of the pattern and the interpolation used to remove the pattern rotation to make this comparison. However, an optimised feature detection stage would ideally select these stronger features to ensure robustness to rotational changes. Clearly, an improved form of feature detection that is robust to decorrelation of the speckle pattern due to translations and rotations is required.

### 5.4. Feature Description Speed

The processing time required for the calculation of the descriptor for each of the different feature matching methods described in Table 2 was investigated using Data Set 2 (Table 3). The results are shown in Table 6, where the average times taken to compute descriptors for 1000 features are listed. Here it can be seen that the computation of the ORB descriptor is significantly faster than for the other methods. Both USURF and BRISK have comparable processing times; however, USURF takes no account of the feature orientation. SURF is the slowest descriptor to compute due to the need to compute a rotated grid around the feature. It can also be seen that the descriptor options (see Table 2), descriptor length (USURF and SURF), and patch size (ORB and BRISK) have relatively little influence on the processing time requirements.

### 5.5. Feature Matching Robustness

Similar to the above analysis for detectors, the robustness of the different feature matching methods was investigated. This will again be important to minimise computation cost and allow reliable measurement with smaller images and hence fewer features. However, when considering the overall robustness, there are three issues: (1) the robustness of the orientation calculation used to guide the descriptor calculation, (2) the robustness of the calculated descriptor to changes in the recorded speckle pattern, and (3) the discrimination between valid matches and random feature pairs. These will result in either a failure to match or incorrect matches and will reduce the measurement range and accuracy achievable. These are discussed in the following sections.

#### 5.5.1. Orientation Robustness

**Figure 10:**
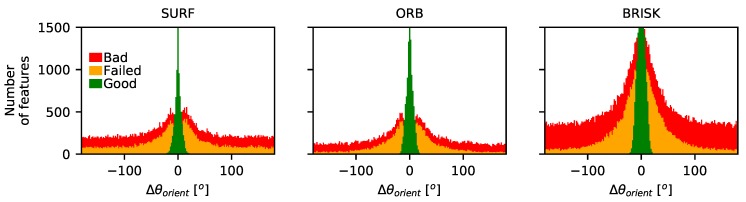
Histogram showing the numbers of features successfully matched (good), those incorrectly matched (bad), and those that failed to match (failed) versus the difference in the calculated feature orientation (after correction for applied rotation). Results shown here are for the stepped rotation data set using the flat lapping reaming ($R_{z}$ = 1.6 µm) surface finish; however, similar results are found for other surfaces.

The first factor that will influence the overall performance of the description and matching stage is the robustness of the orientation calculation used to guide the descriptor. If the orientation angle is not repeatably calculated as the speckle pattern translates, rotates, and decorrelates, then the descriptors calculated will be different even for an identical speckle, resulting in unsuccessful matching. To investigate this, a set of matching features present in two translation-corrected windows can be found using the proximity test method described in Section 5.1. The descriptors are then calculated for these points using the different approaches detailed in Table 2, before being matched using the brute force matching method [19] and the appropriate distance measure. The results of this process can then be divided into three subsets: features matched successfully (‘good’), features matched incorrectly (‘bad’), and features that failed to match (‘failed’), as determined using the proximity test described in Section 5.1. The differences between the orientation angles calculated for the same feature in different windows (after correcting for any known image rotation) can then be examined. The results can be seen in Figure 10, where the differences in calculated orientation for the same feature between the two windows are shown as histograms for the different methods (excluding the USURF method in which no orientation calculation is performed) and the flat lapping reaming ($R_{z}$ = 1.6 µm) stepped rotation data set. Here, the number of features that failed to match or that matched incorrectly are shown stacked as red and orange bars, while successfully matched features are shown in green. Similar results are seen for the other surface finish samples and for translating samples.

**Figure 11:**
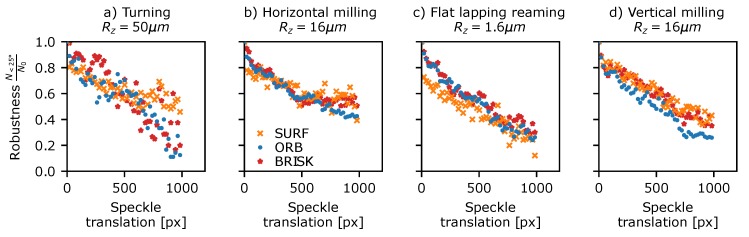
Orientation robustness plotted against measured speckle translation for a selection of surface treatments shown in Figure 1. (**a**) Turning $R_{z}$ = 50 µm, (**b**) horizontal milling $R_{z}$ = 16 µm, (**c**) flat lapping reaming $R_{z}$ = 1.6 µm, and (**d**) vertical milling $R_{z}$ = 16 µm.

**Figure 12:**
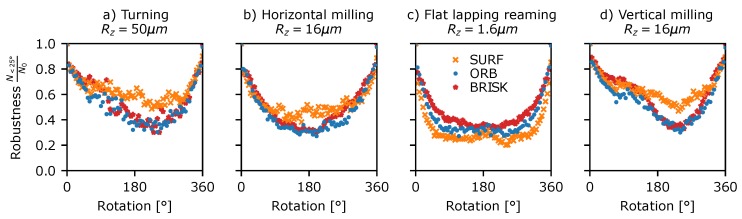
Orientation robustness plotted against rotation angle for a selection of surface treatments shown in Figure 1. (**a**) Turning $R_{z}$ = 50 µm, (**b**) horizontal milling $R_{z}$ = 16 µm, (**c**) flat lapping reaming $R_{z}$ = 1.6 µm, and (**d**) vertical milling $R_{z}$ = 16 µm.

From these results it can be seen that features that matched successfully all have differences in the calculated orientation of <25°, while bad or unmatched features have orientation differences that are more widely distributed. Although the histograms show the combined results of all rotation angles and hence show large numbers of bad and failed matches, it was also observed that even at small translations and rotations a significant number of features are failing to match. This is shown in Table 7 where the percentages of features that were successfully matched, incorrectly matched and failed to matched are shown for a 100 pixel translation and 10° rotation. Not all of these failures can be attributed to the miscalculation of the orientation angle; however, as no features are successfully matched when the difference in the calculated feature orientation is >25°, the robustness of the orientation calculation can be defined by the ratio $R_{orient.}=N_{<25{}^{\circ}}/N_{0}$, where $N_{<25{}^{\circ}}$ is the number of features with the difference in calculated orientation between the two windows of <25°, and $N_{0}$ is the number of matching features found by the proximity test. The values of $R_{orient.}$ are shown in Table 7. Also included are similar results for the USURF method, which does not perform any orientation calculation but rather assumes a fixed feature orientation, so $R_{orient.}=1$. However, it can be seen that USURF matches significantly more features than the SURF method, and this highlights the influence of this failure mode and suggests that, in applications where only small rotations are expected, using an unorientated method may be preferable. However, for larger rotations, the calculation of the orientation will be an essential step (see Section 5.5.2 on descriptor robustness below). Figure 11 and Figure 12 show the change in $R_{orient.}$ with the translation and rotation of the speckle pattern, respectively. Similar plots for the other surfaces can be found in Supplementary Materials. From this it can be seen that the number of matchable features will drop rapidly and that the three methods of calculating the orientation perform generally similarly with some differences between the speckle patterns recorded from different surface finishes. Methods of improved orientation calculation, as defined by $R_{orient.}$, would allow greater numbers of matching features and an extended measurement range and is the second area identified for future improvement.

#### 5.5.2. Descriptor Robustness

**Figure 13:**
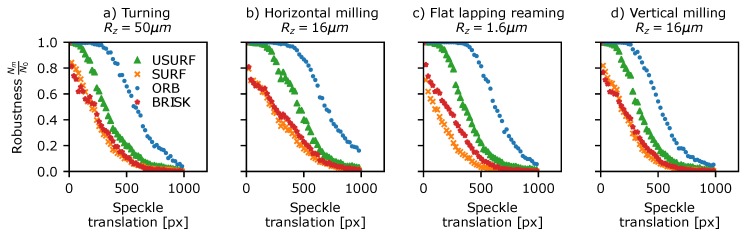
Descriptor translation robustness plotted against measured speckle translation for a selection of surface treatments shown in Figure 1. (**a**) Turning $R_{z}$ = 50 µm, (**b**) horizontal milling $R_{z}$ = 16 µm, (**c**) flat lapping reaming $R_{z}$ = 1.6 µm, and (**d**) vertical milling $R_{z}$ = 16 µm. Here, the USURF & SURF methods use the standard descriptor length of 64 values, and ORB and BRISK are using 32 × 32 pixel patch sizes.

The second issue to consider is the robustness of the calculated descriptor, i.e., the ability of the descriptor to match features even in the presence of small changes to the speckle pattern. Such changes may result from rotations, translations, and the associated decorrelation of the pattern along with changes in the spatial sampling of the speckle field. To investigate the descriptor robustness to translational changes, a set of translation-corrected windows was again extracted from the stepped translation data (Data Set 4, Table 3). Features were then detected in the reference window with all features within a 32 pixel border region discarded to ensure descriptors using image patch sizes surrounding the feature of up to 64 × 64 pixels could be calculated. The descriptors were then calculated at the same positions and with the same orientation, in the reference window and subsequent shifted windows, using the different approaches detailed in Table 2. The brute force matching method [19] was then applied using an appropriate distance measure. This method ensures that the descriptor comparison is independent of orientation calculation and that there are sufficient matchable features present between windows. The resulting matches are then validated by a proximity test, and the descriptor matching robustness is calculated as $R_{descrip.}=\frac{N_{m}}{N_{0}}$, where $N_{m}$ is the number of successfully matched features, and $N_{0}$ is the number of features originally found. The results are shown in Figure 13 for a selection of surface finishes (see Supplementary Materials for other surfaces). Here it can be seen that the robustness reduces with increasing translation, with the best performing method ORB having only approximately 50% of features matched after 500 pixels translation. The USURF method is the next best performing, and both BRISK and SURF perform similarly. The cause of the unorientated version of the SURF descriptor performing better than the orientated version is probably due to the influence of ‘bad’ matches, which is discussed further in Section 5.5.3.

**Figure 14:**
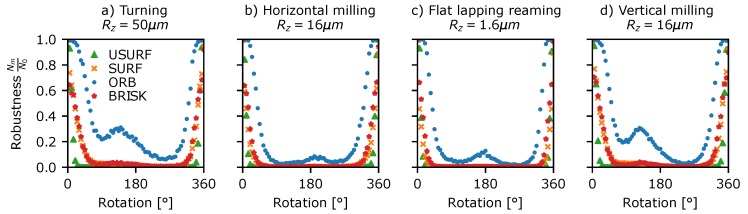
Descriptor rotational robustness plotted against applied rotation for a selection of surface treatments shown in Figure 1. (**a**) Turning $R_{z}$ = 50 µm, (**b**) horizontal milling $R_{z}$ = 16 µm, (**c**) flat lapping reaming $R_{z}$ = 1.6 µm, and (**d**) vertical milling $R_{z}$ = 16 µm. Here, the USURF & SURF methods use the standard descriptor length of 64 values, and ORB and BRISK are using 32 × 32 pixel patch sizes.

A similar method was applied to investigate rotational robustness. A set of circularly masked translation-corrected windows centred upon the rotation were extracted from the stepped rotation data (Data Set 5, Table 3). Features are then detected in the reference windows inside a circular region of radius 360 pixels, and the descriptors are calculated. The co-responding features and their descriptors in the rotated windows are then found by first rotating the key-point coordinates by the known stage rotation and adjusting the key-point orientation value accordingly, before calculation of the descriptor. These are then matched and validated, and the robustness is calculated as before, with the results shown in Figure 14 for a selection of surface finishes (see Supplementary Materials for other surfaces). Again, the ORB method performs the best; however, the proportion of successfully matched features drops rapidly with an increasing rotation angle. There is a slight increase at 180° rotation when the spatial sampling of the speckle is effectively the same but flipped. The asymmetry in the robustness for some samples can be explained by lower levels of in-plane error motion of the stage at these rotations, as observed in the process of extracting the translation-corrected windows. This leads to less decorrelation in the pattern and hence better matching performance. The SURF and BRISK methods again both perform similarly, while the USURF method, having no feature orientation calculation, unsurprisingly performs the worst, with the proportion of matched features falling to ∼10% by the 15 operating angle quoted by the authors [27].

#### 5.5.3. Descriptor Discrimination

**Figure 15:**
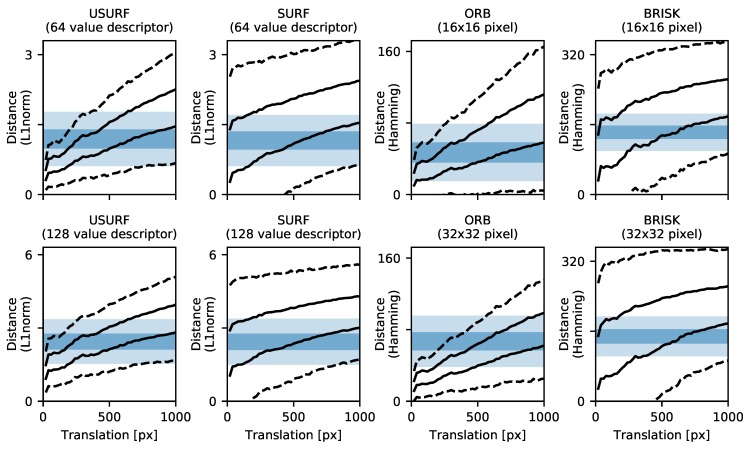
Descriptor discrimination for translation of the horizontal milling $R_{z}$ = 16 µm sample. The shaded bands show the 1 and 3 standard deviation spread in the descriptor distances for ‘bad’ matches. The lines show the 1 and 3 standard deviation spread in the calculated distances for valid matches after translation.

**Figure 16:**
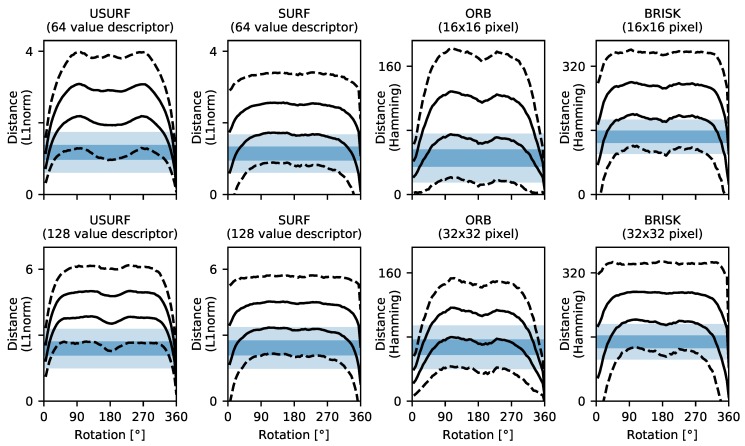
Descriptor discrimination for rotation of the horizontal milling $R_{z}$ = 16 µm sample. The shaded bands show the 1 and 3 standard deviation spread in the computed descriptor distances for ‘bad’ matches. The lines show the 1 and 3 standard deviation spread in the calculated distances for valid matches after rotation.

The final consideration is the discrimination of the descriptor, i.e., how the typical descriptor distance (L1-norm, L2-norm, or Hamming distance) between a pair of matching features compares to the distance between an incorrectly matched pair and can be used to explain the results above. If the typical distance for such a ‘bad’ match is not far from or overlaps with that of a valid match, then the matching process is likely to fail, reducing the usable information and hence requiring more features/larger image sizes and more processing overhead. As the speckle pattern decorrelates with displacement and/or rotation, the distances between valid matches are also likely to increase. To investigate this, the distances of successfully matched pairs (as determined using the proximity testing method above) were compared with the distances of ‘bad’ matches resulting from matching 1000 features with a set of uncorrelated features. The results are shown in Figure 15 and Figure 16 for varying translations and rotations, respectively. Here, the shaded bands show the 1σ (standard deviation) and 3σ bands around the mean distance of the ‘bad’ matches, and the lines show the 1σ and 3σ bands around the mean distance of successfully matched features. The greater the separation between these two bands, the greater the discrimination of the descriptor and the higher the proportion of successfully matched features.

These results help to explain the descriptor robustness results of Figure 13 and Figure 14 and the initial performance tests in Section 4. Methods having a greater numbers of matches with distances below the shaded band will result in fewer bad matches and more successful matches. The ORB method has the greatest separation/least overlap between the bands for both translations and rotations and likewise has the best overall descriptor robustness and measurement range. The USURF method performs similarly to the ORB method for translations, but poorly for rotations, where it can be seen that the valid match band quickly increases to above that of a random match. The orientated version SURF and the BRISK method show similar separations between the random and valid match bands, and have a similar performance in terms of descriptor robustness. The selection of the distance threshold described in Section 3.3 that is applied to remove incorrect matches can be guided by these plots, with the threshold set to exclude 99.7% of ‘bad’ matches, i.e., the lower limit of the shaded 3σ band in Figure 13 and Figure 14. For example, a distance threshold of 0.5 was used for the USURF and SURF methods, while the threshold found using this discrimination analysis would be 0.6.

Also shown in Figure 15 and Figure 16 are the results of using different descriptor options or variants. For the USURF and SURF methods, the ‘extended’ option allows the calculation of a longer (128 versus 64 value) descriptor. Although the use of the 128 value descriptor increases the level of the ‘bad’ match band, it does not appear to increase discrimination, as the valid match band also increases and the intersection of the two remains approximately fixed. Therefore, there seems to be no advantage in using the extended descriptor for speckle pattern processing. The ORB and BRISK methods both allow the image patch used to calculate the descriptor to be varied in size (via the ‘patchSize’ option for ORB and ‘patternScale’ option for BRISK). In Figure 15 and Figure 16, the results of using a 16 ×16 and 32 × 32 pixel patch are shown. For the ORB method, moving from 16 ×16 to 32 × 32 pixels raises and narrows the ‘bad’ match band, and it also narrows the valid match band, increasing the discrimination. Larger patch sizes of 48 ×48 pixels and 64 × 64 pixels led to further but less pronounced improvements, but are not shown here for clarity. However, for small image sizes, using a large patch size leads to excessive feature loss in the image border regions where the descriptor can no longer be calculated; hence, a patch size of 32 pixels is recommended. For the BRISK method, the influence of the patch size was less pronounced, with only a slight narrowing of the bands but no increase in discrimination.

#### 5.5.4. Assessment of Overall Feature Matching Robustness

The calculation of the three measures described above—orientation robustness, descriptor robustness, and descriptor discrimination—offer a quantitative means of assessing the performance of a feature matching algorithm for laser speckle processing. They also highlight the main failure modes: failure in the orientation calculation stage and false or ’bad’ matches due to poor descriptor discrimination. From this, it can be seen that an improved description method is desirable—especially to improve the rotation measurement range, which is currently limited both by poor feature orientation robustness and poor descriptor discrimination at angles >25°.

## 6. Conclusions

The potential of feature tracking approaches for applications in laser speckle sensing has been investigated, and the performance has been shown to be comparable to that of the cross-correlation processing conventionally used in laser speckle instrumentation. Comparison of experimental speckle images processed using normalised cross-correlation (NCC) and 3-point Gaussian peak fitting show that feature tracking approaches have good agreement with the correlation-based processing with errors between 0.01 and 0.2 pixels for the four methods investigated. An investigation of bias errors also shows a translation accuracy similar to the normalised cross-correlation approach, with a peak bias error/pixel-locking of ∼0.033 pixels for the NCC method, ∼0.04 pixels using the BRISK method, and 0.025 pixels for the USURF and SURF methods, which is better than that of the NCC method. The ORB method alone performs worse, with peak errors of 0.17 pixels due to the integer pixel location of key points in the method, as opposed to sub-pixel locations for the other methods, something which can easily be modified in a practical implementation. The image size used, and hence the number of features found, was shown to influence both processing time and precision, with a standard deviation of the error between 0.02 and 0.09 pixels for a 256 × 256 image size and processing times from 8 ms (ORB) to 37 ms (BRISK), which are acceptable for many applications.

The main advantage of the feature tracking approach in comparison to cross-correlation-based processing is in the simultaneous measurement of image rotation. The achievable performance was assessed with experimental speckle patterns acquired with a known stage rotation with errors of <0.01° shown to be achievable over a limited angular range of ±10°. Of the feature matching methods tested, the ORB method performed best, allowing for reliable measurements over ±25°. The measurement range and error level was also shown to be limited by the number of successfully matched features, with a typical standard deviation of the error between 0.02 and 0.08° for a 256 × 256 image size.

The results of this initial investigation suggest that increasing the number of successfully matched features is critical to allow increased measurement range and lower uncertainty as well as to allow faster processing using smaller images. An in-depth analysis of the feature detection and description stages was conducted (Section 5) to identify the most suitable feature tracking methods for use with laser speckle patterns, and to identify areas requiring future improvement. As part of this, reference data sets have been acquired with controlled rotation and translations applied by motion stages. Speckle images were acquired from a variety of surface material finishes and different surface roughnesses to allow performance assessment with a variety of different speckle pattern appearances. These are available for the testing of new feature tracking algorithms tailored for laser speckle pattern processing.

Finally, quantitative approaches of assessing feature tracking algorithms have been described. A quantitative means of assessing feature detection methods in terms of detector robustness, $R_{detect}$, has been described and used to assess the different methods investigated in this study. Here $R_{detect.}$ is the proportion of features detected both before and after a speckle pattern translation or rotation and the resulting pattern decorrelation and sampling differences. All methods tested show large losses in features when undergoing translation or rotation, with the best performing feature detection methods being those based upon accelerated segment tests (e.g., FAST and AGAST). These methods show $R_{detect.}$ dropping to 25–30% after a ∼500 pixel translation and a pattern correlation co-efficient of C=0.6. For rotations, $R_{detect.}$ drops to 50% by ∼12° and 10–20% by 90°. Future work should look at developing more robust feature detection methods to increase the proportion of features available for matching. This will allow measurements without processing excessive numbers of features and/or avoid the requirement for larger image sizes, allowing shorter processing times.

Similarly, three measures of assessing the feature description and matching methods have been described. The feature orientation robustness $R_{orient.}$ is the proportion of features whose orientation is computed to within 25° despite speckle pattern translation or rotation and decorrelation. This feature orientation angle is used in the calculation of the descriptor, and it was shown (Section 5.5.1) that successful matching requires that this angle is computed to <25° for all methods tested. It was also shown that the inability to compute the feature orientation reliably is a major source of bad or failed matches, with $R_{orient.}$ dropping to ∼50% for translations of 500 pixels and rotations of ∼90°. Feature orientation methods that are more robust and tailored to laser speckle patterns are required, and this is the second area identified for future work.

The descriptor robustness, $R_{descrip.}$, describes the proportion of features matched successfully in the presence of pattern changes due to decorrelation, independently of the feature orientation calculation. This, together with the descriptor discrimination, the ability to distinguish a ‘bad’ match from a valid match, can be used to assess the performance of the feature description methods. Of the methods tested, ORB was shown to be the most robust, matching ∼60% of features after a 500 pixel translation or a ∼45° rotation, and has the best discrimination between valid matches and ‘bad’ matches of all the methods tested. However, there is still a significant loss of potential information in terms of unmatched or incorrectly matched features; hence, improved description methods tailored to laser speckle patterns require further investigation.

## Figures and Tables

**Table 1:** Summary of feature detection methods, OpenCV python implementation, and parameters used.

Detector Method	Arguments
**Harris corners** **/Good features to Track (GFTT)** *Implementation:* cv2.GFTTDetector	qualityLevel = 0.01 maxCorners = 5000 minDistance = 1 blockSize = 3 useHarris = True (Harris corners) False (GFTT) Harris_k = 0.04
**FAST** *Implementation:* cv2.FastFeatureDetector	threshold = 70% image mean intensity nonmaxSuppression = True type = cv2.FastFeatureDetector_TYPE_9_16
**AGAST** *Implementation:* cv2.AgastFeatureDetector	threshold = 70% image mean intensity nonmaxSuppression = True type = cv2.AgastFeatureDetector_OAST_9_16
**Difference of Gaussians (DoG)** **(USURF detector stage)** *Implementation:* cv2.xfeatures2d.SURF	hessianThreshold = 20 nOctaves = 1 (No image scaling) nOctaveLayers = 3 (default) upright = True (do not compute orientation of feature)
**Orientated FAST** **(ORB detector stage)** *Implementation:* cv2.ORB	fastThreshold = 70% of image mean edgeThreshold = 32 pixels nFeatures = 5000 (set high to prevent capping) scoreType = FAST_SCORE
**Orientated FAST** **(BRISK detector stage)** *Implementation:* cv2.BRISK	thresh = 70% of image mean octaves = 0 (single scale)
**Orientated DoG** **(SURF detector)** *Implementation:* cv2.xfeatures2d.SURF	hessianThreshold = 20 nOctaves = 1 (No image scaling) nOctaveLayers = 3 (default) upright = False (compute orientation of feature)

**Table 2:** Summary of feature description methods, OpenCV python implementation, and parameters used.

Matching Method	Arguments
**USURF** *Detection*: Difference of Gaussian *Description*: 4D/8D Vector of Haar wavelet sums *Implementation*: cv2.xfeatures2d.SURF	hessianThreshold = 30–300 nOctaves = 1 nOctaveLayers = 3 (default) extended = False (64 element descriptor) extended = True (128 element descriptor) upright = True Distance measure = L1-norm Distance threshold = 0.5
**SURF** *Detection*: Difference of Gaussian *Description*: 4D/8D vector of Haar wavelet sums *Implementation*: cv2.xfeatures2d.SURF	hessianThreshold = 30-300 nOctaves = 1 nOctaveLayers = 3 (default) extended = False (64 element descriptor) extended = True (128 element descriptor) upright = False Distance measure = L1-norm Distance threshold = 0.5
**ORB** *Detection*: orientated FAST *Description*: rotated BRIEF (Binary 256bit) *Implementation*: cv2.ORB	fastThreshold = 70% of image mean edgeThreshold = 32 pixels nFeatures = 5000 (set high to prevent capping) scoreType = FAST_SCORE nLevels = 1 (single scale) scaleFactor = 1.2 (default) firstLevel = 0 (default) patchSize = 16, 32, 48, 64 (pixels) WTA_K = 2 points Distance measure = Hamming Distance threshold = 30
**BRISK** *Detection*: orientated FAST *Description*: rotated BRIEF (Binary 512bit) *Implementation*: cv2.BRISK	thresh = 70% of image mean octaves = 0 (single scale) patternScale = 0.43, 0.86, 1.29, 1.73 (16, 32, 48 & 64 pixels) Distance measure = Hamming Distance threshold = 60

**Table 3:** Summary of experimental data sets.

Data Set	Description
1: Continuous translation	A set of 100 512 × 512 pixel speckle patterns from a cast aluminium plate during a continuous linear translation with a velocity of ∼5 mm/s applied in the x-direction. Used to assess translation performance.
2: Uncorrelated speckle patterns	A set of 100 uncorrelated speckle patterns 512 × 512 pixel in size from a cast aluminium plate with no translation or rotation applied. Used for simulated translation/rotation tests.
3: Stepped rotation	A set of 512 × 512 pixel speckle patterns from a cast aluminium plate with in-plane rotations applied between 0 and 360° in 0.5° steps. Used to assess rotation performance.
4: Surface finishes: stepped translation	A set of 1280 × 1024 pixel speckle patterns from the different surface preparation samples shown in Figure 1 with a linear translation between 0 and 2.8 mm in 50 um steps applied in the x-direction. Used to assess detector and descriptor robustness.
5: Surface finishes: stepped rotation	A set of 1280 × 1024 pixel speckle patterns from the different surface preparation samples shown in Figure 1 with in-plane rotations applied between 0 and 360°. Used to assess detector and descriptor robustness.

**Table 4:** Comparison of the measurement precision, the number of features matched ($N_{m}$), and processing times using an Intel i5-4590 CPU (dt) for different window sizes. One standard deviation errors ($\sigma_{A_{x}}$, $\sigma_{A_{y}}$, and $\sigma_{\theta }$) are calculated for translation measurement via comparison with normalised cross-correlation results and zero rotation, and for rotation measurements ($\sigma_{\theta }$ only) by comparison with applied stage rotation.

Method	Translation					Rotation		
**(Image Size)**	$\sigma_{A_{x}}$	$\sigma_{A_{y}}$	$\sigma_{\theta }$	$N_{m}$	**dt**	$\sigma_{\theta }$	$N_{m}$	**dt**
	**(px)**	**(px)**	**(°)**	**(-)**	**(ms)**	**(°)**	**(-)**	**(ms)**
USURF (128, 128 px)	0.06	0.08	0.04	52	3.7	0.22	22	5.0
SURF (128, 128 px)	0.08	0.09	0.05	25	6.8	0.09	16	7.1
ORB (128, 128 px)	0.27	0.28	0.16	28	1.0	0.37	12	1.0
BRISK (128, 128 px)	0.06	0.06	0.04	81	5.4	0.22	25	5.3
USURF (256, 256 px)	0.02	0.03	0.01	310	25.0	0.02	104	20.5
SURF (256, 256 px)	0.03	0.04	0.01	149	43.7	0.05	48	36.4
ORB (256, 256 px)	0.06	0.09	0.02	267	8.1	0.08	58	7.8
BRISK (256, 256 px)	0.02	0.02	0.01	504	36.9	0.06	141	35.4
USURF (512, 512 px)	0.01	0.01	0.00	1361	317.1	0.03	385	153.8
SURF (512, 512 px)	0.02	0.02	0.00	649	445.0	0.05	145	230.3
ORB (512, 512 px)	0.04	0.06	0.00	1384	191.6	0.08	255	104.1
BRISK (512, 512 px)	0.01	0.01	0.00	2333	430.0	0.03	472	280.2

**Table 5:** Average processing time per frame on an Intel core i5-4590 CPU for various methods of feature detection together with mean number of features detected for different image sizes.

Method	Mean Processing Time			Mean Number of Features		
**(Image Size)**	**(ms/frame)**			**(-)**		
	**(128 × 128)**	**(256 × 256)**	**(512 × 512)**	**(128 × 128)**	**(256 × 256)**	**(512 × 512)**
Harris corners	0.39	1.95	8.51	181	547	1552
GFTT	0.55	2.63	11.44	648	2537	5000
FAST	0.11	0.41	1.84	274	1138	4636
AGAST	0.29	1.15	5.16	294	1220	4965
Difference of Gaussian (DoG)	0.68	3.14	14.24	98	499	2230
Orientated FAST (ORB)	0.22	0.78	3.13	274	1138	4558
Orientated FAST (BRISK)	1.33	7.81	43.44	67	681	3826
Orientated DoG (SURF)	1.32	6.25	26.91	98	499	2230

**Table 6:** Average processing time per feature on an Intel core i5-4590 CPU for various methods of feature description.

Method	Description Time
	(ms per 1000 Features)
USURF (length 64 descriptor)	14.9
USURF (length 128 descriptor)	15.7
SURF (length 64 descriptor)	34.1
SURF (length 128 descriptor)	34.8
ORB (patchSize = 16)	1.5
ORB (patchSize = 32)	1.5
ORB (patchSize = 48)	1.5
BRISK (patchSize = 16)	12.2
BRISK (patchSize = 32)	13.8
BRISK (patchSize = 48)	15.8

**Table 7:** Percentage of features matching successfully (good), those incorrectly matched (bad), and those that failed to match (failed) for the different descriptor methods, a 100 pixel translation, and a 10° rotation. Results shown here are for the flat lapping reaming ($R_{z}$ = 1.6 µm) surface finish.

Method	Translation 100 Pixels				Rotation 10°			
	**%good**	**%bad**	**%failed**	$R_{\mathrm{orient}}$	**%good**	**%bad**	**%failed**	$R_{\mathrm{orient}}$
USURF ^1^	89	2	9	1.0	40	23	38	1.0
SURF	46	21	33	0.77	26	30	44	0.62
ORB	56	13	31	0.81	48	18	34	0.76
BRISK	68	9	23	0.79	49	18	33	0.72

^1^ No orientation calculation in the USURF method, hence $R_{orient}=1.0$.

## Supplementary Materials

All data supporting this study are openly available from the Cranfield University repository at http://dx.doi.org/10.17862/cranfield.rd.7673279.
